# Low diffusion capacity of the lung predicts pneumothorax and chest drainage after CT-guided lung biopsy

**DOI:** 10.1186/s13104-022-06234-6

**Published:** 2022-12-01

**Authors:** Espen Asak Ruud, Sigurd Heck, Knut Stavem, Vidar Søyseth, Jon Terje Geitung, Haseem Ashraf

**Affiliations:** 1 Department of Imaging, Akershus University Hospital, University of Oslo, Sykehusveien 25, 1478 LØrenskog, Norway; 2 University of Oslo, Oslo, Norway; 3 Department of Pulmonary Medicine, Akershus University Hospital, Lørenskog, Norway

**Keywords:** CT-guided lung biopsy, Chest tube, Diffusion capacity, Lung function, Pneumothorax, Multivariable analysis.

## Abstract

**Objectives:**

Complications after CT-guided lung biopsy is a burden both for the individual patient and for the overall healthcare. Pneumothorax is the most common complication. This study determined the association between lung function tests and pneumothorax and chest drainage following CT-guided lung biopsy in consecutive patients in a large university hospital.

**Results:**

We prospectively registered 875 biopsy procedures from 786 patients in one institution from January 27th 2012 to March 1st 2017 and recorded complications including pneumothorax with or without chest drainage. Lung function data from 637 patients undergoing 710 of the procedures were available. The association of lung function measures with pneumothorax with or without chest drainage was assessed using multivariable logistic regression analyses. Diffusion capacity for carbon monoxide (DLCO) below 4.70 mmol/min/kPa was associated with increased occurrence of pneumothorax and chest drainage after CT guided lung biopsy. We found no association between FEV_1_, RV and occurrence of pneumothorax and chest drainage. We found low DLCO to be a risk factor of pneumothorax and chest drainage after CT-guided lung biopsy. This should be taken into account in planning and performing the procedure.

## Introduction

Patients with opacities in the lung parenchyma represent an import challenge in pulmonary medicine. Such aberrations may represent infectious, inflammatory but foremost neoplastic processes. At our department, we usually start with a broad examination of these patients, including blood tests and lung function tests such as spirometry and gas diffusion test. However, a morphological diagnosis is essential. Subsequently, tissue samples are often crucial to get a correct diagnosis. If the pathologic opacity cannot be reach by bronchoscopy, CT-guided transthoracic needle biopsy (TNB) is frequently necessary.

TNB of the lung is a well-established and accurate method to acquire tissue for diagnosing pulmonary lesions [1–5]. The most frequent indications for the procedure is to acquire tissue samples for determination of malignancy or guiding oncological treatment in established malignant disease. It also plays a role in acquisition of samples in infectious diseases. An increased incidence and prevalence of lung cancer and widespread use of CT of the chest have led to the detection of more pulmonary nodules than before, and hence an increased demand for the procedure [6, 7].

The overall complication rate of CT-guided TNB is high [8]. Pneumothorax and bleeding in the lung parenchyma are the most common complications [1] with an incidence of 19–60% for pneumothorax [2, 9–17] and 4–9% for pneumothorax requiring chest drainage [10–12, 14, 15, 17].

Most studies on predictors of pneumothorax after CT-guided TNB have a retrospective design and have presented mainly univariable analyses[18, 19]. The biopsy method varies between studies[8] and also within studies, for example in the use of core-biopsy needle vs. fine-needle aspiration[14, 18], coaxial vs. non-coaxial technique[20] and the caliber of the biopsy needle[14, 21].

Emphysema is a predictor of pneumothorax after the procedure in some studies [9–11, 13, 14, 16, 19, 22]. It is also a risk factor of chest drainage after the procedure [4, 10, 14, 22]. To our best knowledge, few studies have determined the association between pulmonary function test (PFT) variables and pneumothorax or chest drainage following CT-guided TNB. One study reported forced expiratory volume in one second (FEV_1_) to be a predictor of pneumothorax [23]. Another study on diagnostic accuracy and complication rates following ultrathin needle (25G) aspiration lung biopsy reported that FEV_1_, FEV_1_ fraction of forced vital capacity (FEV_1_/FVC), forced expiratory flow at 25% and 75% expired volume of forced vital capacity and diffusion capacity of the lung for carbon monoxide (DLCO) was associated with pneumothorax, but only in univariable analysis [24]. To our knowledge no studies have performed multivariable analyses investigating the association between PFT’s including DLCO and pneumothorax after CT-guided TNB. DLCO is a widely used parameter for assessing quality of lung parenchyma in clinical practice [25]. The most common reason for decreased values is emphysema.

Most studies on predictors of complications after CT-guided TNB have mainly been focused on lesion characteristics and technical aspects of the procedure[3, 5, 8, 10, 14, 17]. However, prior to referral to the procedure, patients are evaluated clinically by the pulmonologist with PFT’s to assess the overall preprocedural risk of complications. FEV1 is commonly used to risky stratify patients before lung biopsy procedure. This study aimed to determine the association of PFTs including DLCO with pneumothorax and chest drainage in a large prospective series of CT-guided TNBs.

## Materials and methods

### Sample and study design

All CT-guided TNB’s of pulmonary lesions performed at Akershus University Hospital from January 27th 2012 to March 1st 2017 were prospectively registered using a paper form. A total of 875 procedures were performed in 786 patients.

We had no definite exclusion criteria. Each patient was evaluated individually by the referring pulmonologist and a thoracic radiologist based on clinical findings and PFT data. Some of the cases were also discussed in a multidisciplinary team including thoracic surgeons in addition to pulmonologists and a thoracic radiologist. Lesions in the mediastinum and chest wall were not included. PFTs including measurement of DLCO were performed routinely as part of the investigation prior to the TNB procedure. PFT data were available for 637 patients undergoing 710 CT-guided TNB procedures; 61 patients had two procedures, 6 had three procedures. PFT data including height and weight were retrospectively registered in the study database.

A letter with information was sent to the patients prior to the procedure. Further information was given by a nurse in the referring department and in the CT laboratory by the physician performing the procedure. The study was accepted by the Regional Ethical Committee (REC South East) and the local data protection authority.

### CT-guided TNB procedure

Antitussive and mild sedative drugs (5 mg dihydrocodeine and 5 mg diazepam per os) were administered according to a standard procedure at our institution by the referring department before the procedure. Five different thoracic radiologists with varying degree of experience, from no experience to > 5 years at study start, performed the procedures. Inexperienced radiologists were supervised by a more experienced colleague.

Most of the procedures were performed on a Philips Brilliance 64 or a Philips Inginuity 128 Core, both with a tiltable gantry. A few procedures were done using a Philips ICT 256 with a fixed gantry (Philips, Amsterdam, Netherlands).

In most cases a low-dose CT scan with the patient in the position decided during planning was performed immediately before the procedure. This scan became part of the routine May 20th 2014. Prior to January 2013, a CT scan with multiple single sections of the thorax was routinely acquired after the procedure. A full volume low-dose CT scan after the procedure was part of the routine on subsequent biopsies.

Under intermittent CT fluoroscopy guidance, and using a step-wise technique, a 17 G coaxial needle was inserted and positioned, usually at the peripheral margin of the lesion in question. Access to some of the lesions made it necessary to tilt the CT gantry. For guidance we used single CT slices with a thickness of 5, 7.5 or 10 mm depending on the lesion size, respiratory movement and available scanner options. We did not give instructions on pattern of breathing before or during the procedure. Consequently the patients did not perform breathhold. Final positioning of the coaxial needle was performed at patients’ end-expiration during normal respiration. A reusable core (cutting) biopsy system with 18 G needle and 15 or 22 mm stroke length (Bard® Magnum®, Bard Medical, Covington, GA, USA) was used, and we usually performed three or four needle insertions through the coaxial needle.

The patients had to lie flat in bed in the referring department for 4 hours after the procedure. They were not allowed to eat or drink in this period. A chest X-ray (frontal projection, patient upright) was performed approximately 2 hours after the procedure.

**Fig. 1:**
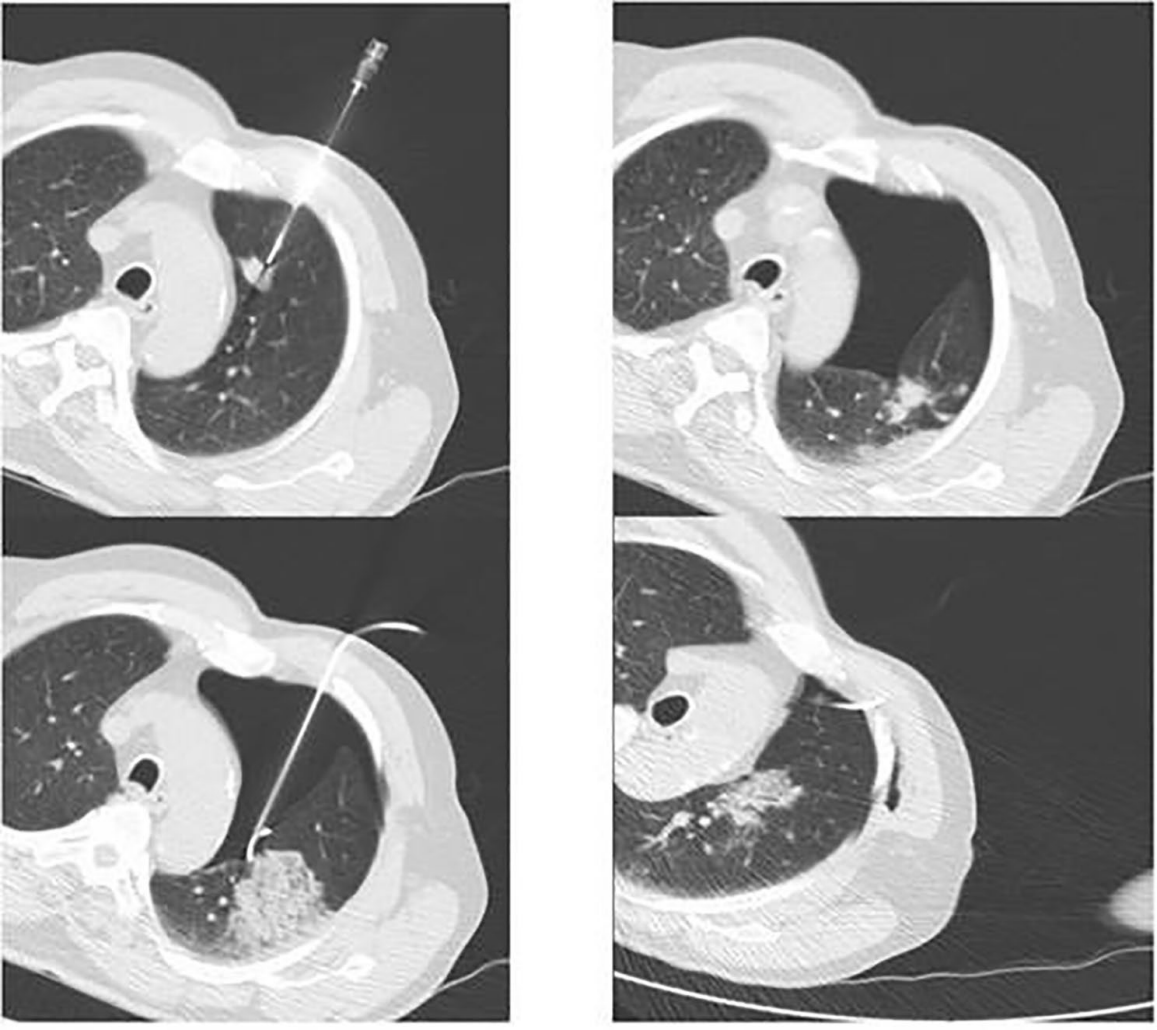
CT guided transthoracic needle biopsy from a lesion in left lungs upper lobe followed by pneumothorax and parenchymal hemorrhage, insertion of a pigtail catheter and aspiration of air

Chest drainage was performed when a pneumothorax occupied more than 20–30% (Fig. 1) of the hemithorax, progressed on the chest X-ray 2 hours after the procedure or was symptomatic. Chest drainage procedures were performed either by the performing radiologist or a thoracic surgeon. Insertions of pigtail catheters and short-term aspirations were performed by the radiologist in the CT laboratory immediately after the procedure. Short term aspirations were done using a thin plastic catheter or a pigtail catheter that was removed before the patient left the CT laboratory. We used pigtail catheters with a French size (FR) of 10 or 12. Chest drainage procedures performed after the patients had left the CT laboratory were performed by thoracic surgeons using Tru-Close Thoracic Vent® (UreSil, Skokie IL, USA) or chest tubes with a size of 22 or 24 FR. Tru-Close Thoracic Vent is a device for drainage of pneumothorax containing a one-way valve eliminating the need for external suction[26].The PFTs in our study consisted of spirometry and a measurement of DLCO. These procedures are part of the routine workup of pulmonary lesions. Spirometry and DLCO measurement were carried out as recommended by American Thoracic Society and European Respiratory Society [27, 28]. Briefly, the spirometry results were given as forced vital capacity (FVC), FEV_1_ and the FEV_1_/FVC ratio. DLCO measurements were accepted provided that the inspiratory volume was 90% or more of the FVC. The PFTs were performed using a Masterscreen PFT® system and Spirometri Sentry Suite version 2.17 (Vyaire Medical, Mettawa, IL, USA).

### Variables and classification

The performing physician registered the procedures on a paper form. The PFT data were collected from the computerized medical record and registered in a database later on by one of the performing physician or a medical student under supervision by the same physician. The baseline data on patients age and sex and lung function parametres are presented in Table 1.

**Table 1:** Baseline characteristics, patients and lung function parameters

Patients	
** Age (mean), (range)**	68.0(22.4-91.7)
≤69.99Years	n=400(56%)
≥70.00Years	n=310(44%)
**Sex (no of patients)**	
Female	n=330(52%)
Male	n=303(48%)
**Lung function parameters**	
**DLCO (ml/min/kPA), mean (range)**	5.89(1.58 – 13.87)
≥6.53	n=236(33%)
4.71 – 6.52	n=238(34%)
≤4.70	n=236(33%)
**FEV**_**1**_ **(litres), mean (range**	2.13(0.48 – 5.29)
≤1.68	n=229(32%)
1.69 – 2.38	n=238(34%)
≥2.39	n=243(34%)
**RV (litres), mean (range)**	2.26(0.81 – 4.50)
≤1.90	n=241(34%)
1.91 – 2.43	n=232(33%)
≥2.44	n=237(33%)

### Registration of pneumothorax and drainage

Any amount of air in the pleural cavity on CT immediately after the procedure and/or on chest X-ray approximately 2 hours after the procedure was registered as pneumothorax. Only one type of chest drainage was registered in each case. When more than one type of drainage was performed, we registered the final type of drainage.

### Statistical analysis

Descriptive statistics are presented with the mean, range or number (%), as appropriate. Some patients had more than one CT-guided TNB. To account for correlation between procedures in the same patients, we analyzed predictors of pneumothorax and chest drainage using a generalized logistic linear mixed model with random intercept.

First we calculated the crude incidence of pneumothorax and chest drainage by covariates and the corresponding odds ratio (OR) and 95% confidence interval (CI). The covariates included in the analyses were DLCO, FEV_1_, RV, age and sex.

PFT variables were categorized as tertiles, which we thought might be clinically relevant, and we calculated ORs for each group accordingly. Age was similarly dichotomized according to age above and below 70 years of age. The results are presented with ORs and 95% CIs.

We selected a 5% significance level, using two-sided tests. Stata version 16.1® (StataCorp, College Station TX, USA) was used for all analyses.

## Results

Pneumothorax occurred in 40% of the procedures, and chest drainage was performed in 10% of the procedures (Table 2). An overview of the patients with pneumothorax vs. no pneumothorax and lung function and age is shown in Table 3, and a similar overview of the patients with chest drainage vs. no chest drainage is shown in Table 4.

**Table 2:** Pneumothorax and chest drainage, overview

Pneumothorax, number (fraction in %)	285/710	(40%)
Chest drainage, number (fraction in %)	74/710	(10%)

**Table 3:** Pneumothorax vs. no pneumothorax and lung function parameters and age

Parameters		Pneumothorax (n=285)	No-pneumothorax (n=425)
DLCO	(ml/min/kPa, mean)	5.57	6.05
FEV_1_	(litres, mean)	2.06	2.16
RV	(litres, mean)	2.26	2.25
Age	(years, mean)	68.6	67.4

**Table 4:** Chest drainage vs. no chest drainage and lung function parameters and age

Parameters		Chest drainage (n=74)	No chest drainage (n=636)
DLCO	(ml/min/kPa, mean)	4.80	5.98
FEV_1_	(litres, mean)	1.94	2.14
RV	(litres, mean)	2.24	2.26
Age	(years, mean)	68.6	67.8

In multivariable analysis, the tertile with lowest values for DLCO (≤ 4.70 mmol/min/kPa) had increased odds of pneumothorax compared to the tertile with highest values (≥ 6.53 mmol/min/kPa) with OR 1.71 (p = 0.04). The tertile with lowest values for DLCO also had an increased odds of chest drainage compared to the tertile with the highest values with OR 6.91 (p < 0.001). FEV_1_ and RV were not associated with pneumothorax or chest drainage, neither was sex. Patient and PFT variables and rate of pneumothorax and chest drainage are presented in Table 5.

**Table 5:** The incidence of pneumothorax and chest drainage after transthoracic lung biopsy by relevant covariates using logistic regression for panel data

			Pneumothorax			Chest drainage	
Covariates	n (%)	OR	95% CI	p	OR	95% CI	p
**DLCO (ml/min/kPa)**							
≥ 6.53	236 (33%)	1			1		
4.71–6.52	238 (34%)	1.40	0.91–2.17	0.127	1.65	0.74–3.66	0.219
≤ 4.70	236 (33%)	1.71	1.02–2.85	0.040	6.91	2.96–16.16	< 0.001
**FEV1 (litres)**							
≤ 1.68	229 (32%)	1			1		
1.69–2.38	238 (34%)	0.89	0.59–1.33	0.569	0.96	0.51–1.81	0.909
≥ 2.39	243 (34%)	1.01	0.63–1.63	0.962	1.96	0.90–4.23	0.088
**RV (litres)**							
≤ 1.90	241 (34%)	1			1		
1.91–2.43	232 (33%)	0.96	0.65–1.41	0.829	1.11	0.59–2.07	0.746
≥ 2.44	237 (33%)	1.09	0.74–1.61	0.672	1.48	0.80–2.76	0.214
**Age**							
≤ 69.99	400	1			1		
≥ 70	310	0.93	0.67–1.30	0.668	0.57	0.33–0.98	0.041
**Sex**							
Male	340	1			1		
Female	370	1.00	0.73–1.36	0.997	1.01	0.62–1.66	0.964

There was no difference in rate of pneumothorax between the group under and above 70 years of age, but individuals > 70 years of age had decreased odds of chest drainage after the procedure with OR 0.57 (p = 0.041).

## Discussion

The major finding in this study was that the incidence of pneumothorax and chest drainage following CT- guided TNB was significantly associated with DLCO. In contrast, FEV_1_ and FVC were not associated with pneumothorax or chest drainage in multivariable analysis.

DLCO is an index of the total alveolar surface area available for gas exchange in the lungs. Measurement of DLCO requires a dedicated procedure in addition to regular spirometry, but this is part of the routine workup before CT-guided TNB in most cases. DLCO is usually reduced in emphysema, alveolar inflammation or pulmonary fibrosis, but low values may also occur in pulmonary embolism, severe cardiac failure, pulmonary hypertension or anaemia[25, 29]. DLCO may be increased in polycythemia, asthma, intracardiac shunting, mild cardiac insufficiency and alveolar bleeding[25, 29]. We believe that the dominant factor affecting DLCO in the population of patients undergoing CT-guided TNB is emphysema.

This study has shown that low values (≤ 4.70 mmol/min/kPa ) for DLCO were associated with increased odds of pneumothorax and chest drainage after CT-guided TNB. Compared to pneumothorax (OR 1.71), patient with DLCO in the lower tertile had a considerably higher odds for chest drainage following the procedure (OR 6.91).

It is unclear why the association of DLCO with chest drainage seemed stronger than with pneumothorax. One possible explanation is that emphysema, as assumed to be the main cause of low DLCO in our population, implies higher rate of leakage of air into the pleural cavity once a pneumothorax is present and hence leads to a larger pneumothorax. It is also possible that a pneumothorax is more prone to give symptoms in patients with emphysema.

We found that age over 70 years was associated with lower rate of chest drainage. In contrast, there was no difference in the rate of pneumothorax between patients under and over 70 years of age. One possible explanation is that older patients to a higher degree are tired after the procedure, and this may interfere with the patients and the performing physician eagerness to perform an additional procedure. The patients’ motivation to return to their homes the same day as the procedures were performed may also have interfered with the eagerness to perform drainage, and it is possible that this motivation is age dependent.

We found no association of RV or FEV_1_ with pneumothorax or chest drainage. FEV_1_ may be affected in lung parenchyma disorders, but is above all reduced in disorders affecting airways and musculoskeletal system and in some neurological disorders. RV is primarily a parameter to quantify hyperinflation in obstructive lung disease, but may also be increased in neuromuscular disorders. Hence, DLCO is a more specific parameter in assessing lung parenchyma than RV and FEV_1_, which is in accordance with our findings.

FEV_1_ is often the highlighted parameter describing a patient’s lung function in the communication between pulmonologists and radiologists. Our findings suggest that DLCO should be kept in mind as a predictor of pneumothorax when planning and performing CT guided TNB. DLCO was associated with pneumothorax and chest drainage after CT-guided TNB and should be taken into account when planning and performing the procedure and evaluating risk versus benefit for the individual patient.

### Limitations

A limitation of this study is that the PFT was not performed the same day of the biopsy therefore; there could be an up to 2 week interval between the PFT being performed and the biopsy procedure. The biopsies in this study were registered prospectively while the PFT data was retrospectively collected. In addition, all the procedures were performed in one hospital, which strengthens data control, but challenges generalization and external validity of the findings.

## Data Availability

The datasets used and/or analysed during the current study available from the corresponding author on reasonable request.

## Abbreviations

CI: Confidence Interval
COPD: Chronic Obstructive Pulmonary Disease
CT: Computer Tomography
DLCO: Diffusion capacity of the lung for carbon monoxide
FVC: Forced vital capacity
FEV1: Forced expiratory volume in 1 s
FEV1/FVC: FEV1 fraction of FVC
FEV1/FVC%: FEV1 fraction in % of FVC
FR: French size of catheter, three times the catheters diameter in millimetres
G: Gauge, unit for caliber of needle
OR: Odds Ratio
PFT: Pulmonary Function Test
RV: Residual Volume
SD: Standard Deviation
TNB: Transthoracic Needle Biopsy

## References

[1] Deng CJ, Dai FQ, Qian K, Tan QY, Wang RW, Deng B (2018). Clinical updates of approaches for biopsy of pulmonary lesions based on systematic review. BMC Pulm Med.

[2] DiBardino DM, Yarmus LB, Semaan RW (2015). Transthoracic needle biopsy of the lung. J Thorac disease.

[3] Li GC, Fu YF, Cao W, Shi YB, Wang T (2017). Computed tomography-guided percutaneous cutting needle biopsy for small (</= 20 mm) lung nodules. Medicine.

[4] Takeshita J, Masago K, Kato R, Hata A, Kaji R, Fujita S (2015). CT-guided fine-needle aspiration and core needle biopsies of pulmonary lesions: a single-center experience with 750 biopsies in Japan. AJR Am J Roentgenol.

[5] Wang Y, Li W, He X, Li G, Xu L (2014). Computed tomography-guided core needle biopsy of lung lesions: Diagnostic yield and correlation between factors and complications. Oncol Lett.

[6] Bellolio MF, Heien HC, Sangaralingham LR, Jeffery MM, Campbell RL, Cabrera D (2017). Increased Computed Tomography Utilization in the Emergency Department and Its Association with Hospital Admission. West J Emerg Med.

[7] Barta JA, Powell CA, Wisnivesky JP (2019). Global Epidemiology of Lung Cancer. Ann Glob Health.

[8] Heerink WJ, de Bock GH, de Jonge GJ, Groen HJ, Vliegenthart R, Oudkerk M (2017). Complication rates of CT-guided transthoracic lung biopsy: meta-analysis. Eur Radiol.

[9] Wu CC, Maher MM, Shepard J-AO (2011). Complications of CT-Guided Percutaneous Needle Biopsy of the Chest: Prevention and Management. Am J Roentgenol.

[10] Hiraki T, Mimura H, Gobara H, Shibamoto K, Inoue D, Matsui Y (2010). Incidence of and Risk Factors for Pneumothorax and Chest Tube Placement After CT Fluoroscopy–Guided Percutaneous Lung Biopsy: Retrospective Analysis of the Procedures Conducted Over a 9-Year Period. Am J Roentgenol.

[11] Covey AM, Gandhi R, Brody LA, Getrajdman G, Thaler HT, Brown KT (2004). Factors associated with pneumothorax and pneumothorax requiring treatment after percutaneous lung biopsy in 443 consecutive patients. J vascular interventional radiology: JVIR.

[12] Fontaine-Delaruelle C, Souquet PJ, Gamondes D, Pradat E, de Leusse A, Ferretti GR (2017). [Predictive factors of complications during CT-guided transthoracic biopsy]. Rev Pneumol Clin.

[13] Kakizawa H, Toyota N, Hieda M, Hirai N, Tachikake T, Matsuura N (2010). Risk factors for severity of pneumothorax after CT-guided percutaneous lung biopsy using the single-needle method. Hiroshima J Med Sci.

[14] Mills M, Choi J, El-Haddad G, Sweeney J, Biebel B, Robinson L (2017). Retrospective analysis of technical success rate and procedure-related complications of 867 percutaneous CT-guided needle biopsies of lung lesions. Clin Radiol.

[15] Vagn-Hansen C, Pedersen MR, Rafaelsen SR. Diagnostic yield and complications of transthoracic computed tomography-guided biopsies. Danish medical journal. 2016;63(6). 27264940

[16] Zhao Y, Wang X, Wang Y, Zhu Z (2017). Logistic regression analysis and a risk prediction model of pneumothorax after CT-guided needle biopsy. J Thorac disease.

[17] Ruud EA, Stavem K, Geitung JT, Borthne A, Søyseth V, Ashraf H. Predictors of pneumothorax and chest drainage after percutaneous CT-guided lung biopsy: A prospective study. European radiology. 2020. 10.1007/s00330-020-07449-6 33354745

[18] Aktas AR, Gozlek E, Yilmaz O, Kayan M, Unlu N, Demirtas H (2015). CT-guided transthoracic biopsy: histopathologic results and complication rates. Diagnostic and interventional radiology (Ankara. Turkey).

[19] Besir FH, Altin R, Kart L, Akkoyunlu M, Ozdemir H, Ornek T (2011). The results of computed tomography guided tru-cut transthoracic biopsy: complications and related risk factors. Wiener klinische Wochenschrift.

[20] Moreland A, Novogrodsky E, Brody L, Durack J, Erinjeri J, Getrajdman G (2016). Pneumothorax with prolonged chest tube requirement after CT-guided percutaneous lung biopsy: incidence and risk factors. Eur Radiol.

[21] Lim WH, Park CM, Yoon SH, Lim H-J, Hwang EJ, Lee JH, et al. Time-dependent analysis of incidence, risk factors and clinical significance of pneumothorax after percutaneous lung biopsy. 2018;28(3):1328–37. 10.1007/s00330-017-5058-7 28971242

[22] Ozturk K, Soylu E, Gokalp G, Topal U (2018). Risk factors of pneumothorax and chest tube placement after computed tomography-guided core needle biopsy of lung lesions: a single-centre experience with 822 biopsies. Pol J Radiol.

[23] García-Río F, Pino JM, Casadevall J, Gómez L, Atienza JM, Díaz-Lobato S, et al. Use of Spirometry to Predict Risk of Pneumothorax in CT-Guided Needle Biopsy of the Lung. 1996;20(1):20–3. 10.1097/00004728-199601000-00005 8576476

[24] Oikonomou A, Matzinger FR, Seely JM, Dennie CJ, Macleod PJ (2004). Ultrathin needle (25 G) aspiration lung biopsy: diagnostic accuracy and complication rates. Eur Radiol.

[25] Krol K, Morgan MA, Khurana S (2019). Pulmonary Function Testing and Cardiopulmonary Exercise Testing: An Overview. Med Clin N Am.

[26] Kim YP, Haam SJ, Lee S, Lee GD, Joo SM, Yum TJ (2017). Effectiveness of Ambulatory Tru-Close Thoracic Vent for the Outpatient Management of Pneumothorax: A Prospective Pilot Study. Korean J Radiol.

[27] Miller MR, Hankinson J, Brusasco V, Burgos F, Casaburi R, Coates A (2005). Standardisation of spirometry.

[28] MacIntyre N, Crapo RO, Viegi G, Johnson DC, van der Grinten CPM, Brusasco V, et al. Standardisation of the single-breath determination of carbon monoxide uptake in the lung. 2005;26(4):720–35. 10.1183/09031936.05.00034905 16204605

[29] Hegewald MJ (2009). Diffusing capacity. Clin Rev Allergy Immunol.

